# Comparison of the Trapping Efficiency for Tryptic Peptides on Particle-Packed and Micro-Pillar Trap Columns for Proteomics Analyses

**DOI:** 10.3390/proteomes14010010

**Published:** 2026-02-18

**Authors:** Jadranka Miletić Vukajlović, Bojana Ilić, Bella Bruszel, Tanja Panić-Janković, Goran Mitulović

**Affiliations:** 1 Department of Physical Chemistry, Vinča Institute of Nuclear Sciences, National Institute of the Republic of Serbia, University of Belgrade, 11000 Belgrade, Serbia; 2 Institute of Science and Technology, 3400 Klosterneuburg, Austria; 3 Klinik Landstrasse, Zentrallaboratorium mit Blutbank, Juchgasse 25, 1030 Vienna, Austria; 4 Department of Laboratory Medicine, Medical University of Vienna, 1090 Vienna, Austria; goran.mitulovic@meduniwien.ac.at

**Keywords:** trap columns, proteomics, peptide identification, high-performance liquid chromatography

## Abstract

Background: Low-volume trapping columns are essential for sample enrichment, desalting, and injection profile focusing on nano-LC–MS-based proteomics. They enable higher sample loading, improve chromatographic performance, and protect the analytical column by removing salts and contaminants. Recently, monolithic trap columns with micropillar architecture have emerged as alternatives to conventionally packed traps. This study compares the performance of a packed and a micropillar monolithic trap column for the analysis of tryptic peptides. Methods: A tryptic digest of HeLa cell lysate was analyzed under identical LC–MS conditions using both trap types. Peptides were detected at 214 nm and analyzed by nano-ESI on a Q Exactive Plus Orbitrap. Data were searched against the human UniProt database (February 2023) using FragPipe v20.0, and statistical evaluation of MaxLFQ intensities was performed in Perseus using Welch’s *t*-test and clustering analysis. Results: Over 2500 proteins were identified with both setups. The packed trap column yielded more total peptides, particularly those with post-translational modifications and higher hydrophilicity, whereas the monolithic column favored peptides of intermediate hydrophobicity. Chromatographic profiles confirmed a slight reduction in the trapping efficiency of hydrophilic peptides by the monolithic trap. Conclusions: Trap column design significantly influences peptide recovery and proteome coverage.

## 1. Introduction

The sample complexity of tryptically digested proteins necessitates precleaning and desalting to enable proper separation and higher identification rates [1,2,3,4,5,6,7]. HPLC-MS is the most widely used analytical technique for peptide separation, analysis, and identification in proteomics [8,9,10,11,12].

Numerous proteomics studies employed a setup with a trap and an analytical column packed with identical stationary phases—typically C18 material. However, the particles used in these two columns may differ in particle size and, sometimes, in pore size. Unlike in the analytical column’s stationary phase, the particles used for trap columns are usually larger, contributing to lower loading backpressure and faster sample injection. Packed trap and separation columns remain the first choice for peptide separations in nano-LC systems, with fully porous particles dominating applications. Monolithic trap columns and pillar array (μPAC) trap columns are also used [13,14,15,16,17,18,19,20,21,22] but have not yet gained wider acceptance, although their performance can significantly contribute to deeper insight and identification.

The packed-bed capillary column has proven to be the most reliable tool for peptide separation, delivering high peak capacities with manageable back pressure [9,23,24,25,26,27,28]. However, new stationary phases for reverse-phase separation of tryptic peptides are emerging, demonstrating that chromatographic separation can be significantly improved on these columns [27,29,30,31,32,33,34,35,36,37,38,39], resulting in more identified proteins with better amino acid sequence coverage, which, in turn, leads to better quantification.

Depending on the starting material (e.g., tissue or cell culture), the injection volume of a trypsin-digested sample can be as high as 50 µL or more [40,41]. Directly injecting this volume into a nano-separation column would cause peak broadening and poor separation, resulting in fewer identified peptides and, even worse, quantification with high CV values [42].

However, large sample volumes can be directly injected with a low-elution-strength mobile phase when a trap column is employed. Moreover, cleaning the sample and removing residual salt and buffers after enzymatic digestion or multidimensional separation is necessary to protect the separation column from impurities and prevent them from reaching the mass spectrometer.

Although trap columns with inner diameters of 75 µm and 150 µm have been introduced, the 300 µm ID trap column has been widely accepted for nano HPLC and proteomics applications [43]. We anticipate that trap columns with smaller IDs will find wider use in single-cell proteomics and metabolomics analyses, where minimal sample dilution is required to maintain the highest possible sensitivity. Using this method, the sample is preconcentrated, cleaned, and focused on the trap column in a single step, removing the limitations of direct injection. After concentrating on the trap column, analytes are transferred to the separation column with a low-volume switching valve in either the front-flush or back-flush mode. The back-flush method is often preferred because it causes less band broadening than the front-flush mode [43,44,45,46]. However, trap columns without a frit at the column inlet can only operate in front-flush mode.

Trap columns are often avoided due to a “sample and information loss” compared to direct injection. However, if a proper loading mobile phase using a potent ion-pairing agent is employed, only a slight difference between direct sample injection and preconcentration on a trap column is observable. A slight difference in peptide retention times is observed if connections are correctly made due to the increased volume of the separation system. As an example, an adequately installed trap column with a 300 µm inner diameter and 5 mm length, packed with 3 µm particles and 120 Å pores, will generate approximately 1.2–1.8 µL of additional system volume [43].

This manuscript describes the performance of the µPAC trap column, which was tested and compared to our laboratory’s state-of-the-art packed-bed trap column used for peptide separations.

## 2. Materials and Methods

### 2.1. Instrumentation

All chromatographic separation was performed using a nanoRSLC 3000 UltiMate system (Thermo Fischer Scientific, Vienna, Austria).

Before separation, two trap columns were used for sample loading, peptide trapping, and desalting. The PepMap C18 trap column (Thermo Fischer Scientific) with 300 μm ID × 5 mm length, 5 μm particle size, and 100 Å pore size and the μPAC C18 trap column (PharmaFluidics (now Thermo Fisher Scientific), Gent, Belgium) with 2 µm pillar height × 10 mm length, 5 μm pillar diameter, and 100–200 Å pore size were mounted on a ten-port column in the column oven and operated at 50 °C.

The μPAC separation column (PharmaFluidics (now Thermo Fisher Scientific), Ghent, Belgium) was used in all experiments. The separation column features a 5 μm pillar diameter, a 2.5 μm inter-pillar distance, an 18 μm pillar height, a 315 μm bed channel width, and a total column length of 200 cm. The pillars in this separation column are superficially porous, end-capped with hydrophobic C18 chains.

### 2.2. Mass Spectrometry

Mass spectrometry analysis was performed using the Q-Exactive Orbitrap Plus, equipped with a Flex nano-ESI source and a stainless-steel emitter needle (20 µm ID × 10 µm tip ID). The ESI emitter voltage was set to 3.1 kV, and the scan range was 200–2000 *m*/*z*. The full MS resolution was set to 70,000, the AGC target to 3 × 10^6^, and the maximum injection time was set to 50 ms. For the MS/MS analysis, the mass resolution was set to 35.000, the AGC target to 1 × 10^5^, and the maximum injection time to 120 ms. The isolation width for MS/MS was set to *m*/*z* 1.5, and the top 10 ions were selected for fragmentation; single-charged ions and ions bearing a charge higher than +7 were excluded from MS/MS. Dynamic exclusion time was set to 20 s. The NCE was set to 30.

### 2.3. Sample

The performance of the two trap columns for analyzing complex biological samples was tested using a digested protein mixture from HeLa cell lysate (Thermo Fisher Scientific, Vienna, Austria). According to the manufacturer’s instructions, the original content of the HeLa standard was dissolved in 80 μL of aqueous 2% ACN, 0.1% TFA, and 0.01% HFBA. The solution was diluted 2.5-fold with 0.1% aqueous TFA for injection, resulting in 100 ng/µL of digested HeLa proteins. In total, 500 ng (5 µL) of digested HeLa proteins were injected into the RP-HPLC system for analysis.

A total of five injections were performed using each trap column.

### 2.4. HPLC Conditions and Mobile Phases

The mobile phase used for sample loading by the loading pump consisted of 2% ACN, 0.1% TFA, and 0.01% HFBA. The mobile phase was cooled to 3 °C during sample loading, and a flow rate of 30 µL/min was maintained [47]. The trapping duration was set for 2 min, after which the trap column was switched in line with the separation column.

The trap and the separation column were operated at 50 °C in a column oven. Peptides were eluted with a gradient of the following solvents: Mobile phase A (MPA): 95% water, 5% acetonitrile, 0.1% formic acid, and mobile phase B (MPB): 95% acetonitrile, 5% methanol, 0.1% formic acid. Both trap columns were operated in the backflush mode.

Peptide detection was performed using a UV detector at 214 nm and a nano ESI on a Q-Exactive Plus Orbitrap mass spectrometer (ThermoFisher Scientific, Bremen, Germany).

### 2.5. Separation of Complex Biological Sample (PepMap and μPAC)

An identical chromatographic separation gradient profile was applied regardless of the trap column used.

Chromatographic separation was performed at 600 nL/min, using the following gradient: an isocratic phase with 2% B maintained for 10 min, followed by a linear increase in B to 60% over 150 min. The column and trap column were flushed with 90% B for 15 min, then equilibrated for 25 min before the next run. Blank samples (loading solvent injections) were used between samples to clean the system and prevent carry-over.

The trapping time was optimized in a previous study [48]. Briefly, the loading time and flow rate were optimized for sample loading, and the flushing of impurities was optimized based on the trap column’s void volume, injector volume, and the amount needed to wash out potential contaminants from the 300 μm ID trap column packed with particulate stationary phase. The most practical and acceptable trapping time, based on a loading flow of 30 μL/min and a 20 µL sample loop, was 2 min. Identical loading settings were used for both trap columns.

### 2.6. Data Analysis

The first run of the five consecutive runs on each trap column was excluded from statistical analysis due to a shift in retention time. Raw MS data were searched against the human UniProt protein database (version February 2023) using FragPipe 20.0 [49,50,51]. The precursor mass tolerance was set to 20 ppm, and the fragment ion tolerance was also set to 20 ppm. Trypsin (strict trypsin) was specified as an enzyme with up to 2 missed cleavages, and the minimum peptide length was set to 7. Search criteria included carbamidomethylation of cysteine as a fixed modification, with oxidation of methionine, acetylation (protein N-terminus), phosphorylation (STY), ammonia loss, and water loss as variable modifications. The false discovery rate (FDR) was set to 1%, and a decoy-database search was used to estimate it. Match between runs and cross-normalization of intensity were disabled.

Kyte–Doolittle hydrophobicity values were calculated using Peptides (ver. 2.4.4) [52,53].

The heatmap was generated using instatclue v0.12.2 (https://www.instantclue.uni-koeln.de/ (accessed on 2 February 2026))

For the statistical comparison, the Welch t-test was conducted on the MaxLFQ intensities using Perseus (version 1.6.5.0) [54].

## 3. Results

This manuscript explores the optimal approach to achieving maximal trapping efficiency with two advanced trap columns used in proteomics studies. There was no targeted search for proteoforms identified exclusively with one of the configurations applied in this work. A limitation of the current manuscript is that only the standard, commercial HeLa sample was used for testing, with no biological samples included in this setup.

**Figure 1:**
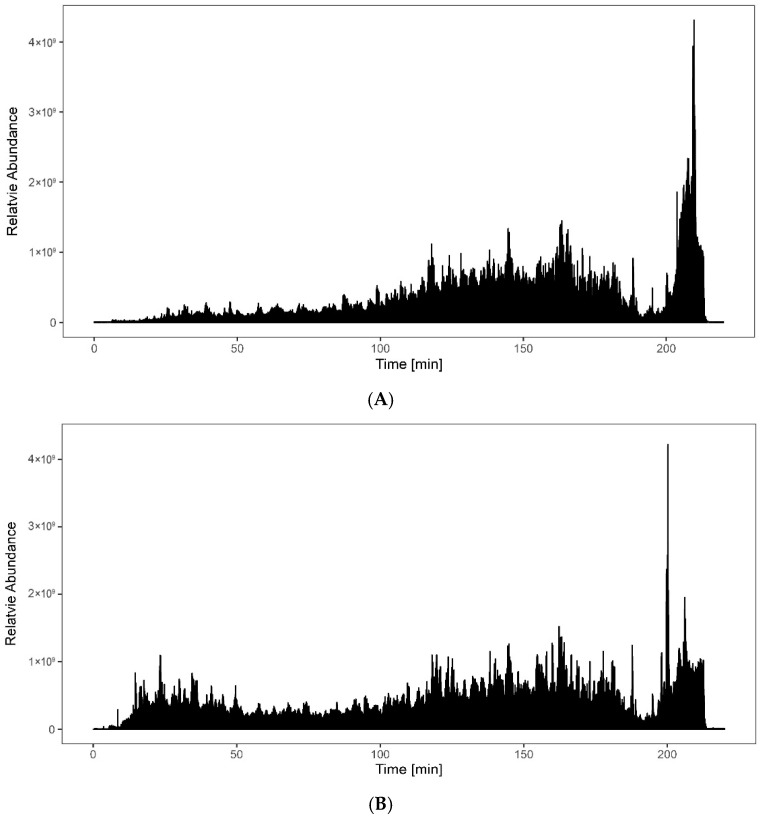
Total ion chromatogram showing separation of 500 ng HeLa tryptic peptides: (**A**) Monolithic trap column (top). (**B**) Packed trap column (bottom). The monolithic trap shows fewer signals in the hydrophilic part of the chromatogram, resulting in fewer identified peptides and lower protein sequence coverage. See also Figure 2 and Figure 3.

More than 2500 proteins were identified using both trap columns in this experiment. However, more peptides were identified with the packed trap column than with the monolithic trap column. The base peak chromatograms for both setups are shown in Figure 1. A slight difference is observed for the early-eluting peptides, but the signal intensities are similar in both setups. The monolithic trap column exhibits slightly denser elution of peptides in the hydrophilic region, indicating stronger trapping of hydrophilic peptides. Search results also confirmed this observation.

**Figure 2:**
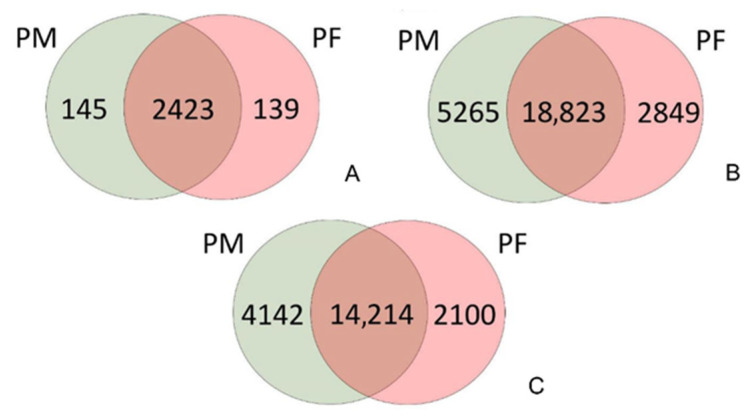
Comparison of identified proteins and peptides for both trap columns. The figure shows the total number of identified peptides and proteins, respectively, from five consecutive injections of digested HeLa using both configurations: PM = PepMap, and PF = µPAC trap columns. (**A**) Number of identified proteins; (**B**) number of identified peptides, including post-translational modifications; (**C**) number of identified peptides, excluding post-translational modifications. For selected PTMs included in the analysis, see Section 2.

In addition to the total number of identified peptides, the packed trap column increases the number of peptides with selected posttranslational modifications (Figure 2), indicating differences in trapping preferences and interactions between peptides and the stationary phase.

**Figure 3:**
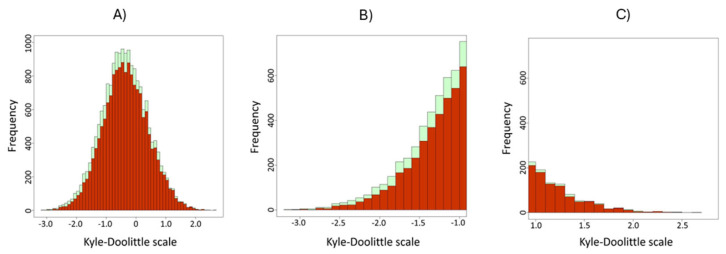
Peptide distribution based on hydrophobicity by Kyle–Dolittle: (**A)** All identified peptides with both trap columns. (**B**) A higher number of hydrophilic peptides is identified using the packed trap column (green) than the monolithic trap column (red). (**C**) The difference in the number of identified hydrophobic peptides with both trap columns is smaller than for the hydrophilic ones.

Figure 3 shows the difference in trapping efficiency depending on peptides’ hydrophobicity on the tested columns under identical experimental conditions. The monolithic trap column did not trap hydrophilic peptides as effectively as the packed trap column, resulting in fewer identifications. The list of all identified peptides and an overview of their hydrophobicity are provided in the Supplemental Tables.

Using different trap columns did not significantly affect the peak capacity of the separation system. For both combinations, peak capacity was calculated using the equation:

$$PC=\frac{t_{g}}{\frac{\sum_{1}^{3}{FWHM}_{first\;peak}+\sum_{1}^{3}{FWHM}_{last\;peak}}{6}}$$

where $t_{g}$ is the length of the gradient slope, and *FWHM* is the peak width at half maximum. The *FWHM* was calculated using Chromeleon (version 6.8) for five consecutive runs.

For the combination of the particulate-packed column and the monolithic separation column, a peak capacity of 870 was obtained, and a peak capacity of 890 was obtained for the combination of the µPAC trap column and the µPAC separation column.

**Figure 4:**
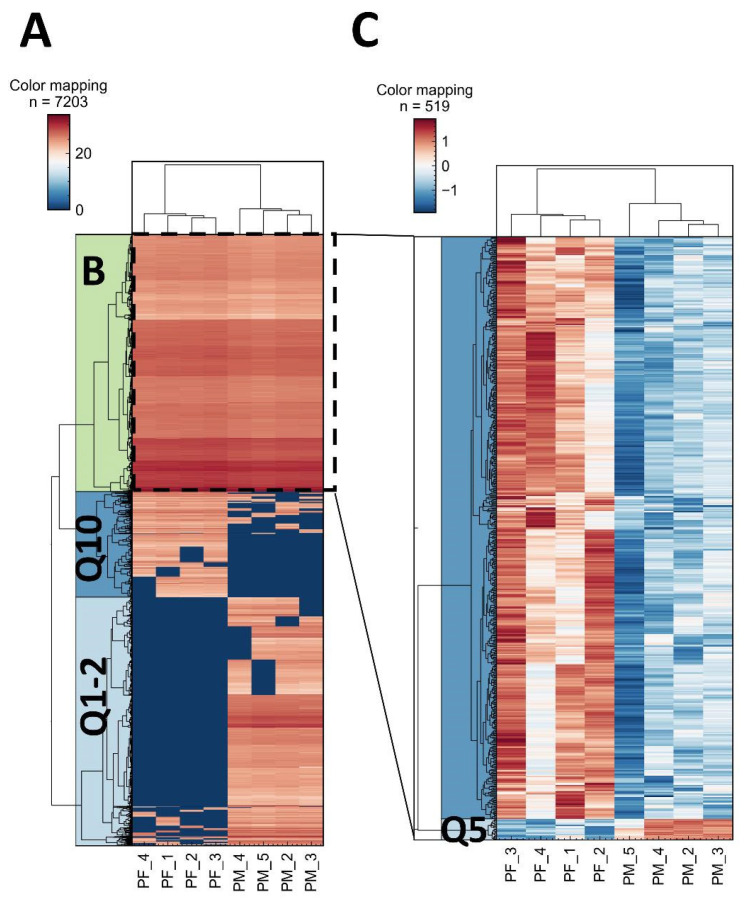
(**A**) Hierarchical clustering of peptide intensities that differed significantly (*p* ≤ 0.05, FDR = 20%, missing values replaced with zeroes, dark blue for PF hydrophilic area (Q1–Q2) and PM hydrophobic area (Q10)) was performed. (**B**) Significantly changed peptides quantified with both trap columns. (**C**) Z-score normalized cluster B for better visualization of differences in peptide quantities on both trap columns, showing stronger retention of hydrophobic peptides on the PM trap column, which corresponds to the results shown in Figure 3.

Figure 4 provides a comprehensive overview of the hierarchical clustering analysis performed to investigate systematic differences in peptide detection between the two trap columns. By grouping peptides with significantly altered intensities, this analysis highlights distinct patterns associated with their physicochemical properties, particularly hydrophobicity. The resulting clusters enable a clearer interpretation of how each trap column influences peptide retention and subsequent identification. Together, these observations offer a more detailed understanding of the analytical behavior of the two workflows and their impact on the overall depth of peptide profiling. Figure A shows that the particulate-packed trap column (PM) retains hydrophylic peptides stronger than the μPAC trap column. However, μPAC trap column traps hydrophobic peptides more efficiently and generates higher signal intensities. The difference was made visible by z-score normalization of cluster B, shown in Figure 4C. Interestingly a small set of peptides from Q5 (middle range hydrophobicity on the Kyte–Doolittle scale) shows higher signal intensity on the particulate-packed trap column (PM).

## 4. Discussion

The use of trap columns became essential in bottom-up proteomics analysis. The trap column removes particulate matter and salts from the injected sample, enabling faster injection times and shorter cycle times. However, selecting the wrong trap column can significantly undermine the analysis, potentially leading to loss of information. Trap columns are mainly used to capture and concentrate analytes before they are separated on analytical columns. The trapping efficiency for hydrophilic and hydrophobic species varies based on the loading mobile phase, ion-pairing agents, operational temperature, and flow rate during sample loading.

Furthermore, the trap column can also be a significant contributor to the system’s void volume. In addition, chromatographic analysis of proteomics samples is always accompanied by sample dilution and, often, a significant increase in the sample volume [39,40]. The sample volume used for direct injection onto a conventional nano-HPLC column with a 75 µm inner diameter is typically limited to 1–2 µL [42], thus excluding larger sample volumes routinely obtained with most biological samples.

From a chromatographic perspective, it is advisable to use a trap column for: (a) Cleaning up the sample. (b) Concentrating the sample when in large volume and a classical solid-phase tip cleaning could lead to a sample loss and generate a narrow injection profile suitable for the separation column’s volume (inner diameter and length). (c) Enabling solvent exchange and removing ion-suppressing agents before LC separation and MS detection. Additionally, the selected trap column should: (a) Be compatible with the separation column in terms of the stationary phase and loading capacity. (b) Exhibit minimal void volume to prevent peak broadening in the extra column.

Another factor that significantly impacts results is that the void volumes in a nano-HPLC system are undesirable because they act as a “mixing chamber,” reducing separation efficiency, decreasing sensitivity, and leading to poor identification and quantification performance.

Capriotti et al. and Simone et al. [55,56] described the separation of tryptic digests of proteomic samples into two fractions based on molecular weight and hydrophobicity using two trap columns. In a recent paper, Zhu et al. described the use of immiscible-solvent sandwich injection for proteomic sample analysis [57]. They examined how immiscible solvents can prevent and suppress axial dispersion and reduce band broadening. Results show that this method significantly improves the peak shape for early-eluting substances. This technique could also be combined with trap columns to compress the injection profile further and minimize peak broadening.

The concept of the µPAC (micropillar array column) separation column enables the practical elimination of axial dispersion and a significant reduction in it, thereby increasing the maximum column plate numbers and achieving sharper peaks. This, again, will contribute to higher peak capacity and, eventually, higher identification numbers of peptides and proteins from complex samples [58,59].

In addition to the µPAC separation column, a µPAC trap column was also introduced. Theoretically, using both the trap and the separation column based on identical technology should be the ideal combination, eliminating potential interferences and incompatibility, such as peak broadening.

Our objective was to compare the chromatographic performance of methods for peptide separation using both trap-column designs and, eventually, to introduce the µPAC trap column for regular use in our laboratory.

Although higher MS signals were observed with the µPAC trap column, more peptides were identified in the hydrophilic region when the packed trap column was used. The increased number of identified proteins and peptides, as shown in Figure 2 and Figure 3, supports this finding. Based on these facts, the lower numbers of identified proteins and peptides can be explained by using the µPAC trap column. Additionally, the previously mentioned study helps explain the lower count of hydrophilic peptides identified with the monolithic column. The higher flow rate during sample loading, the lack of porosity, and the rapid mass transfer on the monolithic trap column result in lower yields. Although identical loading conditions are applied to all peptides, hydrophobic ones exhibit stronger interaction with the stationary phase, and the identification numbers are comparable to those with the packed trap column. Detailed results for each trap column type and run are available as Supplementary Data Tables S1–S4.

Although the number of identified proteins differs slightly across experiments, more pronounced differences are evident at the peptide level. The overall number of identified peptides with the PM trap column is higher, and the number of peptides exclusively identified with the packed trap column is almost double the number of peptides identified when the PF trap column was used.

As the hydrophobicity of the peptides increases, the performance and trapping efficiency of the traps become more similar, and the difference in identified peptides decreases for hydrophobic analytes. The variation in the number of identified peptides can be explained by the fact that monolithic columns or columns packed with superficially porous particles have lower mass capacity, leading to analyte loss due to overloading. For detailed data on how the hydrophobic nature of identified peptides affects the identification rates, please see the Supplementary Table S1.

**Figure 5:**
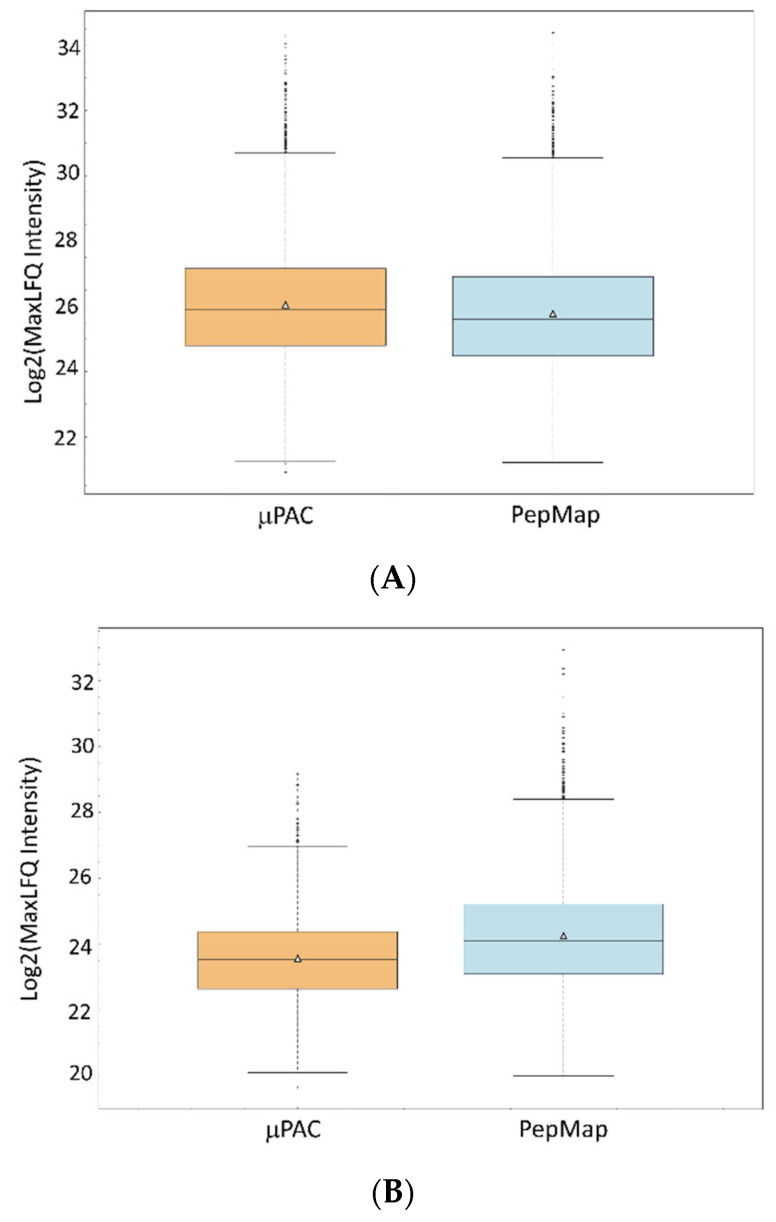
Box plots comparing peptide MaxLFQ intensities across trap column types, including peptides quantified with both columns with at most one missing value in each dataset (**A**), and (**B**) peptides that are exclusively quantified in either the µPAC or the PepMap trap columns in at least two replicates. The PepMap exclusive peptides show higher intensities than the µPAC exclusive peptides (48% median, 60% average). A triangle represents the mean value.

When comparing the intensities of identified peptides, we see differences in MaxLFQ (average) intensities of the shared peptides, Figure 5A. From the results, it looks like the µPAC trap column resulted in 20% higher intensities (the difference is roughly 20% for both median and average) of identified peptides.

In Figure 5B every peptide that was quantified with at least two valid values with the µPAC or the PepMap trap is included, and the MaxLFQ intensities of the trap-exclusive peptides were compared. It can be speculated that the higher intensities correlate with the higher number of peptides identified when the PepMap trap columns were used.

Monolithic columns generally have larger external porosities, allowing more of the mobile phase to flow through the column than packed columns do. Additionally, the pillars of the µPAC column and trap column are only partially porous, reducing the relative amount of stationary phase in the μPAC compared to packed bed columns. The relatively large pores (about 15 nm) reduce the number of available active sites for analyte binding.

Clinical proteomics is gaining significance, and both researchers and clinicians are facing issues of sample throughput. Processing large sample cohorts for both discovery and diagnostic purposes requires fast sample loading and separation. Current methods allow high throughput only by using short columns or high flow rates, both of which affect the depth and reproducibility of the analysis [60,61,62,63]. The use of monolithic micropillar columns and the corresponding trap columns could, in the long term, bridge the gap between speed and chromatographic resolution.

A factor not considered in this study is the sample loading speed. For both trap columns, identical conditions were applied to the sample loadings, without accounting for the monolithic material’s superficial porosity. The rapid binding and release processes on monolithic and superficially porous stationary phases may be further accelerated at high flow rates. However, we did not explore lower flow rates for sample loading at this stage; this will be done in future experiments.

## 5. Conclusions

Both trap columns meet the requirements for separation stability and reproducibility, but they also show significant differences. The packed trap column yielded better results, particularly in the total number of identified peptides and hydrophilic peptides.

Both trap columns can be used with the µPAC separation column. Although it might be assumed that the monolithic trap column would perfectly fit the monolithic separation column, the data tells a different story. The use of a new type of trap column, µPAC traps, demonstrated that these columns can be used for bottom-up proteomics; however, users must be cautious to avoid losing hydrophilic peptides.

## Data Availability

The mass spectrometry proteomics data have been deposited to the ProteomeXchange Consortium via the PRIDE partner repository with the dataset identifier PXD052759.

## Supplementary Materials

The following supporting information can be downloaded at: https://www.mdpi.com/article/10.3390/proteomes14010010/s1, Original figure 1_exported from the Thermo QuanBrowser; Table S1: PepMap peptide merged; Table S2: uPAC peptide merged; Table S3: PepMap protein merged; Table S4: PF protein merged.

## Abbreviations

RP-HPLC	Reversed-phase high-performance liquid chromatography
MS	Mass spectrometry
ESI	Electrospray ionization
SPE	Solid-phase extraction
LLE	Liquid–liquid extraction
PGC	Porous graphite carbon
TFA	Trifluoroacetic acid
PFPA	Pentafluoropropionic acid
HFBA	Heptafluorobutyric acid
PFPA−	Pentafluoropropionate phosphate anion
HFBA−	Heptafluorobutyrate phosphate anion
MP	Mobile phase
FDR	False discovery rate
