# Supplementary material for: Comparison of the Trapping Efficiency for Tryptic Peptides on Particle-Packed and Micro-Pillar Trap Columns for Proteomics Analyses
Source: Proteomes. 2026 Feb 18;14(1):10. doi: 10.3390/proteomes14010010 (PMC13030385; doi:10.3390/proteomes14010010)
Supplement: Supplementary file 1 [file proteomes-14-00010-s001.zip › original figure 1_exported from the Thermo QuanBrowser.pdf]

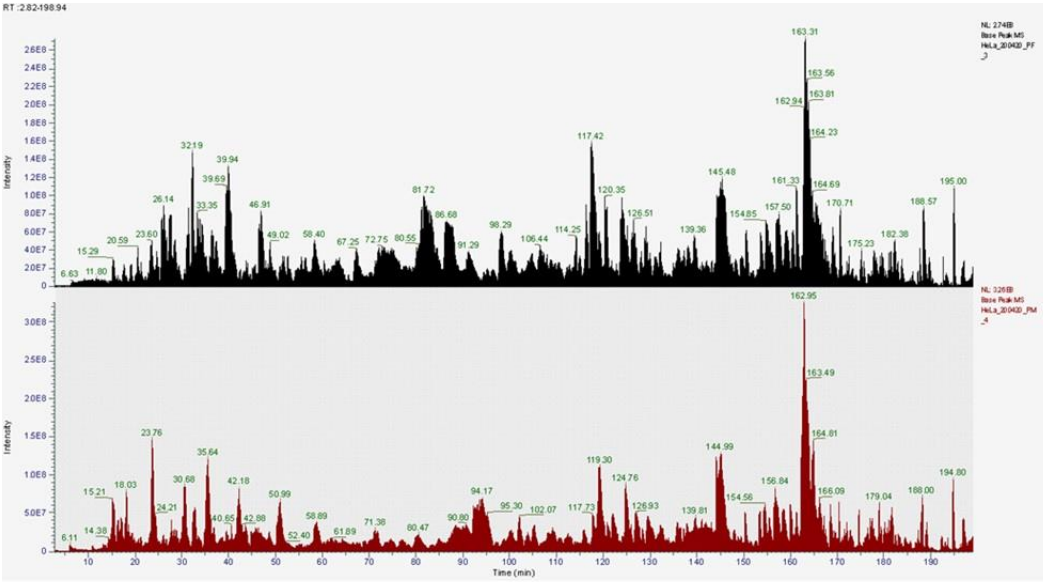

Original figure 1_exported from the Thermo QuanBrowser. This figure was edited for better visibility in the main manuscript.
